# Inhibitory Potential of Boscalid and Abamectin Towards Acetylcholinesterase and Butyrylcholinesterase: Computational and In Vitro Studies

**DOI:** 10.3390/ijms26072865

**Published:** 2025-03-21

**Authors:** Biljana Arsić, Stefan Petrović, Budimir S. Ilić, Milka Vrecl, Tomaž Trobec, Kristina Sepčić, Robert Frangež, Sanja M. Glišić, Jelena S. Milićević

**Affiliations:** 1 Department of Chemistry, Faculty of Sciences and Mathematics, University of Niš, 18000 Niš, Serbia; stefan.petrovic@pmf.edu.rs; 2 Department of Chemistry, Faculty of Medicine, University of Niš, 18000 Niš, Serbia; budimir.ilic@medfak.ni.ac.rs; 3 Institute of Preclinical Sciences, Veterinary Faculty, University of Ljubljana, 1000 Ljubljana, Slovenia; milka.vreclfazarinc@vf.uni-lj.si (M.V.); tomaz.trobec@vf.uni-lj.si (T.T.); robert.frangez@vf.uni-lj.si (R.F.); 4 Department of Biology, Biotechnical Faculty, University of Ljubljana, 1000 Ljubljana, Slovenia; kristina.sepcic@bf.uni-lj.si; 5 Department for Bioinformatics and Computational Chemistry, Institute of Nuclear Sciences, University of Belgrade, 11000 Belgrade, Serbia; sanja@vin.bg.ac.rs

**Keywords:** pesticides, cholinesterase, enzyme inhibition, electron-ion interaction potential, molecular docking, ADMET, molecular dynamics simulations

## Abstract

The growing demand for agricultural products has led to the misuse of pesticides, resulting in the use of higher concentrations of these substances. This has led to an increase in toxicity imposed on other beneficial organisms and to the bioaccumulation of toxic pesticide concentrations in the bodies of both pests and non-target organisms, as well as in their end users, including humans. In this study, the neurotoxic potential of the commonly used pesticides abamectin (an insecticide) and boscalid (a fungicide) was evaluated. Both in vitro and in silico studies showed that human butyrylcholinesterase is not a target for abamectins B1A and B1B. Boscalid showed a modest Glide score (−28.8 kJ/mol) and a considerably higher IC_50_ (308.8 µM) against human butyrylcholinesterase than the approved inhibitor (2-((1-(benzenesulfonyl)-1*H*-indol-4-yl)oxy)ethyl)(benzyl)amine (IC_50_ = 0.473 µM). However, due to its non-mutagenicity and low toxicity, structural analogues of boscalid might be considered as candidates for the symptomatic treatment of Alzheimer’s disease. Molecular dynamics simulations over 100 ns confirmed the stability of boscalid within the active site of butyrylcholinesterase, where it maintained key interactions with catalytic residues such as Trp82 and His438. These findings highlight its potential as a starting point for structure-based drug design strategies aimed at optimizing cholinesterase inhibitors with improved pharmacokinetic properties. According to absorption, distribution, metabolism, elimination, and toxicity studies, boscalid is orally active, which cannot be attributed to abamectins B1A and B1B.

## 1. Introduction

Currently, modern intensive agriculture is faced with serious problems due to residues of chemical pesticides in food and feed, environmental concerns, human health issues, and the rising human population [1,2]. Among these chemical pesticides, abamectin and boscalid are commonly used.

Abamectin is an insecticide of natural origin obtained by the fermentation of the soil bacterium *Streptomyces avermitilis*. It was discovered in the mid-1970s during a screening for natural products with antihelmintic properties [3]. Abamectin targets glutamate-gated chloride channels in the nerve and muscle cells of invertebrates. It increases chloride ion influx, leading to cell membrane hyperpolarization with the disturbance of normal nerve signaling, finally causing the paralysis and death of target organisms. The lack of specific glutamate-gated chloride channels in mammals is the reason for abamectin’s selective toxicity against invertebrates [4]. However, abamectin can also interact with GABA-gated chloride channels in mammals, which may cause neurotoxic effects [5].

Boscalid was developed by BASF in 1995 [6] and is used in agriculture to control molds and fungi. It targets succinate dehydrogenase (SDH), an enzyme of the Krebs cycle and mitochondrial electron transport chain that catalyzes the oxidation of succinate to fumarate. The inhibition of SDH leads to impaired cellular respiration, decreased energy production, and slowed fungal growth [7]. Because SDH is involved in energy metabolism in almost all eukaryotic organisms, boscalid may be harmful to non-target species [8]. Its effects on cholinergic enzymes have not been thoroughly investigated.

To identify promising candidates for new agricultural antifungal agents, Wen et al. (2010) [6] further developed and synthesized a series of boscalid-based nicotinamides. These newly synthesized nicotinamide compounds were tested for their antifungal and insecticidal activities, and some of them were found to be even more effective against the plant pathogenic fungi *Gibberellazeae*, *Rhizoctonia solani*, *Fusarium oxysporum*, *Colletotrichum capsici*, and *Valsa mali* in comparison to boscalid. Further, the replacement of the biphenyl group in boscalid with a more potent diaryl ether scaffold, while retaining the nicotinamide functional group, resulted in the onset of insecticidal activity in some of the tested compounds.

Today, there is an intensive global focus on studying the degradation mechanisms of pesticides and their retention rates (leaching) in soil. Song et al. (2020) [9] investigated the degradation rates of boscalid and abamectin in strawberry samples by treating the fruit with their recommended doses and at twofold the recommended doses of these pesticides. The residues of both pesticides in strawberries showed first-order kinetic degradation. The half-lives of boscalid were between 8.0 and 8.4 days. No abamectin residues were observed at the recommended doses, which was probably due to the low active ingredient content in the formulation and to the additional dilution during application. However, at twofold doses, these residues were detected [9]. Further, researchers from China studied the degradation and leaching of 17 commonly used pesticides, including abamectin and boscalid, in greenhouse soils in Shandong Province to obtain data on their behavior under real conditions and provide a scientific basis for controlling pesticide pollution. The degradation of most of the pesticides, including boscalid, showed second-order kinetics. The half-lives of these pesticides under light and dark conditions ranged from 2.5 to 104 days (average: 36.2) and from 2.6 to 110 days (average: 31.4), respectively. The half-lives of several pesticides, including abamectin B1A, were less than 30 days, indicating their easy degradability. The studied pesticides showed leaching potential in the following descending order: nitenpyram ≫ metalaxyl > acetamiprid > carbendazim > diethofencarb ≈ chlorantraniliprole > isoprothiolane > oxadixyl > boscalid ≈ tebuconazole > hexaconazole. From these data, it can be concluded that boscalid does not belong to the group of pesticides with a high leaching potential, meaning that it can remain in the soil for longer time periods and poses only a low risk of groundwater contamination. In contrast, pesticides that leach easily but degrade slowly, such as chlorantraniliprole and metalaxyl, pose a significant risk of groundwater contamination and require additional technologies to mitigate this environmental risk [10]. Regardless of their degradation and leaching properties, all these chemicals might pose a threat to different non-target organisms, and it is, therefore, important to evaluate various aspects of their toxicity.

In previous studies, we conducted an analysis of pesticides and drugs used for the symptomatic treatment of Alzheimer’s disease and investigated their interaction with acetylcholinesterase (AChE) by molecular docking studies. Acetylcholinesterase is an enzyme that catalyzes the hydrolysis of the neurotransmitter acetylcholine and, thus, regulates its concentration in synapses. The hydrolysis of acetylcholine can also be catalyzed by a related, non-specific cholinesterase—butyrylcholinesterase (BChE), also known as serum cholinesterase or pseudocholinesterase. Under certain pathophysiological conditions, BChE can act as a substitute for AChE in the hydrolysis of acetylcholine. However, AChE exerts significantly broader functions compared to BChE [11]. Due to their ability to increase the concentration of acetylcholine in nerve synapses, reversible ChE inhibitors can be used as drugs for the symptomatic treatment of Alzheimer’s disease [12,13].

The results of our previous study showed that pesticides such as acetamiprid, cymoxanil, chlorantraniliprole, metalaxyl, methomyl, and thiacloprid showed better AChE inhibition compared to rivastigmine, a drug commonly used for the symptomatic treatment of Alzheimer’s disease, while emamectin was inactive in this regard [14]. In this study, we focused on the investigation of the potential inhibition of AChE and BChE by the pesticides boscalid and abamectin using in vitro experimental and in silico methods, in particular molecular docking and molecular dynamics simulations. These studies are the first step towards investigating the possible neurotoxic effects of these pesticides.

## 2. Results and Discussion

Boscalid and components of abamectin (abamectins B1A and B1B) show completely different values of AQVN and absolute EIIP. In the case of boscalid, the AQVN is 2.9142 Ry and the absolute EIIP is 0.0112 Ry, while abamectins B1A and B1B have similar AQVN (2.597 Ry and 2.6107 Ry, respectively) and EIIP values (0.0834 Ry and 0.0810 Ry, respectively). It is, therefore, not surprising that boscalid and abamectins B1A and B1B show different in silico behavior toward AChE, and especially toward BChE. Due to the similarity of the AQVN values of boscalid and chlorantraniliprole (3.0000), similar in silico behavior was expected, and indeed they showed similar Glide scores against hAChE (−20.3 kJ/mol and −19.4 kJ/mol, respectively) [14].

The global minima of selected pesticides (XYZ coordinates are available in the Supplementary Materials) were docked into the overall structure and the binding sites, and the Glide scores are listed in Table 1.

In vitro studies showed that boscalid and abamectins B1A and B1B had no inhibitory effect on human AChE in the concentration range tested (0–500 µM) (Table 2). Docking to the hAChE binding site characteristic of donepezil, a drug used for the symptomatic treatment of Alzheimer’s disease, further showed that abamectins B1A and B1B could not dock. In contrast, boscalid had a better Glide score than donepezil (−20.3 kJ/mol vs. −16.3 kJ/mol, respectively) (Table 1).

Further, human BChE was not shown to be a target for abamectins B1A and B1B both in silico and in vitro (Table 1 and Table 2). This result was expected, as these compounds have similar properties to other 16-membered macrocycles [15]. In contrast, boscalid exhibited a moderate inhibitory activity (IC_50_ = 308.8 µM) against human BChE. Furthermore, a lower Glide score was observed compared to the approved BChE inhibitor (2-((1-(benzenesulfonyl)-1*H*-indol-4-yl)oxy)ethyl)(benzyl)amine (−28.8 kJ/mol vs. −36.1 kJ/mol, respectively). It is, therefore, not surprising that its IC_50_ was considerably higher than that of the above-mentioned inhibitor (IC_50_ = 0.473 µM) (Table 2) [16,17,18].

Molecular docking can be used as a valuable tool for predicting the activity of compounds against both human cholinesterases. In a previous study, abemectin was determined as a reversible, competitive inhibitor of horse serum BChE in both enzymatic and in silico studies [19]. However, in our in vitro tests (Table 2), abamectin failed to inhibit the same enzyme. At present, the reasons for this observed lack of activity in our study are unknown. The differences might result from the use of different enzyme lots or from the use of different buffers for the kinetic assays (phosphate buffer in our case and MOPS (3-(*N*-morpholino)propanesulfonic acid) in the study by Terali et al. (2018) [19]), which can influence the activity of cholinesterase enzymes [20].

Theoretical calculations of the ADMET parameters using QikProp are shown in Table 3 along with Lipinski and Jorgensen rule violations. Based on these results, boscalid seems to be orally active, which cannot be attributed to abamectins B1A and B1B. ADME and drug safety profiling (Table 4) further showed that boscalid, despite having a better Glide score than donepezil, is a weak central nervous system penetrant according to Percepta, so it is not an appropriate candidate for the symptomatic treatment of Alzheimer’s disease. However, since it is non-mutagenic and has a low toxicity (>5000 mg/kg) [21], it could be redesigned to achieve better central nervous system penetration. Abamectins B1A and B1B were found to be non-mutagenic compounds with non-penetrating properties to the central nervous system (Table 4).

Following molecular docking, in vitro inhibition assays, ADMET and ADME analyses, metadynamics simulations, and molecular dynamics (MD) simulations were conducted to determine the preferential binding conformation and further investigate the ligand’s binding stability and thermodynamic preference. Since boscalid was the only compound exhibiting in vitro inhibitory activity (Table 2), it was selected for further analysis. To distinguish between the two docking poses of boscalid within human butyrylcholinesterase (hBChE), metadynamics simulations were performed. The results demonstrated that the binding pose within the active site (−35.8 kJ/mol) exhibited a lower free energy and was, therefore, more thermodynamically stable than the whole-structure docking pose (−32.5 kJ/mol). To validate these findings, MD simulations were conducted to evaluate boscalid’s stability within the active site and to characterize its key interactions with critical residues. Throughout the 100 ns simulation, the root-mean-square deviation (RMSD) and root-mean-square fluctuation (RMSF) values for boscalid remained below the 2 Å threshold, confirming the structural stability of the complex [22].

**Figure 1:**
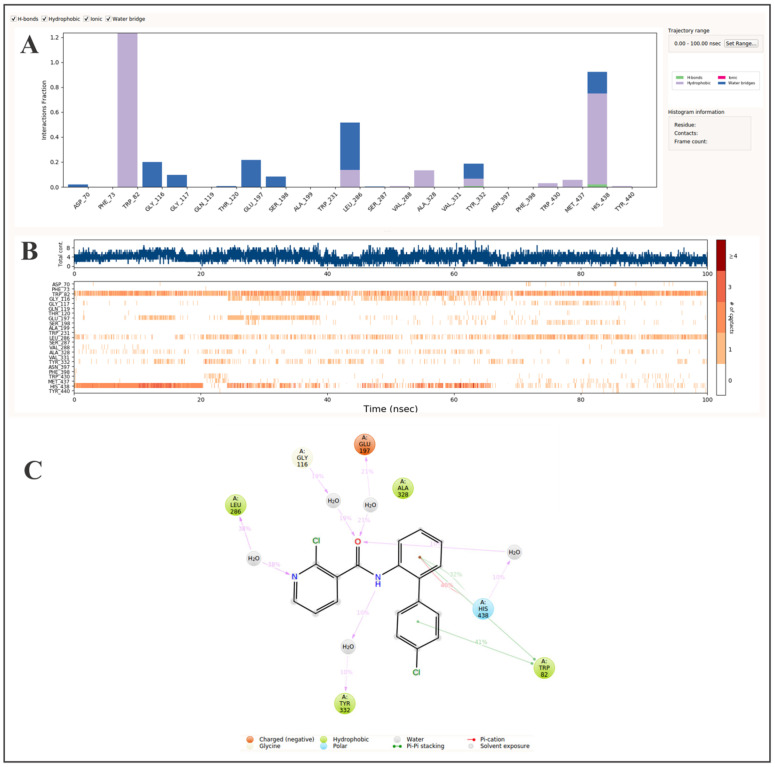
Molecular dynamics analysis of boscalid binding to butyrylcholinesterase over 100 ns: frequency distribution of interactions (**A**), timeline of key interactions (**B**), and spatial contact map (**C**).

Previous studies have highlighted the significance of several amino acid residues in the active site gorge of hBChE, including Trp82, Gly116, Glu197, Leu286, Tyr332, and His438, which play critical roles in ligand binding and enzymatic inhibition [23,24]. Consistent with previous findings, our MD simulations confirmed that boscalid maintained stable interactions with these key residues throughout the simulation, further supporting its binding affinity and stability within the active site (Figure 1). The molecular dynamics simulations demonstrated that π-π stacking with Trp82 was the key interaction stabilizing boscalid within the hBChE active site, aligning with the primary binding mechanism of phenothiazine [25]. Since phenothiazine also predominantly interacts with Trp82, this shared interaction pattern explains the comparable IC_50_ values of the two compounds, with boscalid at 308.8 μM and phenothiazine at 137 μM, further reinforcing the alignment between the computational predictions and experimental inhibition data.

Additionally, the benzene ring and the carbonyl oxygen of boscalid established interactions with the catalytic residue His438 for 92% of the simulation time. Among these interactions, hydrophobic contacts were the most prevalent, followed by water-bridged interactions, while hydrogen bonding was the least frequent (Figure 1). In comparison, MD simulations of ondansetron also confirmed interactions with His438 [26]; however, unlike boscalid, ondansetron formed additional key interactions with the critical residues Trp231 and Phe329, further enhancing its binding stability [23]. These interactions contribute to its stronger binding affinity and greater inhibitory potency, as evidenced by a binding free energy of −54.14 kJ/mol and an IC_50_ value of 2.5 μM, indicating a stronger hBChE inhibitory effect than boscalid [26].

Furthermore, the acyl-binding pocket residue Leu286 remained in contact with the pyridine ring of boscalid for 52% of the simulation time, predominantly through water-bridged interactions, with weaker contributions from hydrophobic interactions (Figure 1). Similarly, the choline-binding pocket residue Glu197 and the oxyanion hole residue Gly116 established water-bridged interactions with the carbonyl oxygen of boscalid, persisting for 22% and 20% of the simulation time, respectively. Additionally, Tyr332 contributed to ligand stabilization via water-bridged interactions with the amide nitrogen of boscalid, accounting for 19% of the simulation time, along with minor hydrophobic and hydrogen bonding contributions (Figure 1).

These findings collectively underscore the stability and specificity of boscalid’s interactions within the hBChE active site, with π-π stacking with Trp82 as the dominant binding mechanism, while additional hydrophobic and water-bridged interactions enhance its moderate inhibitory potency and binding affinity.

## 3. Materials and Methods

### 3.1. Cholinesterase Inhibition Assay

The activities of the ChEs were determined using a modification of the Ellman method [27] adapted for microtiter plates [28]. Stock solutions of the potential inhibitors abamectin (Sigma-Aldrich; PESTANAL^®^, analytical standard; remarks on HPLC ≥ 95.0% (sum of abamectins B1A and B1B)) and boscalid (both Sigma-Aldrich, St. Louis, MO, USA) were prepared in 100% MeOH (2 mg/mL). A positive control (2 mg/mL neostigmine methyl sulfate; Tokyo Chemical Industry Co., Ltd., Tokyo, Japan) was also prepared in pure MeOH. The stock solutions of the potential inhibitors, the positive control, and the negative control (MeOH) were added to the wells and progressively diluted in 100 mM potassium phosphate buffer (pH 7.4) to a final volume of 50 μL. Then, 100 μL of acetylthiocholine chloride (1 mM) and 5,5′-dithiobis-2-nitrobenzoic acid (0.5 mM) in 100 mM potassium phosphate buffer (pH 7.4) were added into the microtiter plate wells. Human recombinant AChE (hAChE), horse serum BChE (hsBChE) (all Sigma-Aldrich, St. Louis, MO, USA), and human recombinant BChE (hBChE) (gift from research group of Professor Stanislav Gobec, Faculty of Pharmacy, University of Ljubljana) were dissolved in the same buffer to a final concentration of 0.0075 U/mL. In total, 50 μL of each ChE solution was added into the microtiter plate wells to start the reaction, which was followed spectrophotometrically at 405 nm at 25 °C over 5 min using a kinetic micro-plate reader (Dynex Technologies Inc., Chantilly, VA, USA). Blank reactions without the inhibitors were run with the appropriate dilutions of the solvents in which the tested compounds were initially diluted (MeOH), and the readings were corrected according to the appropriate blanks. Each measurement was carried out in three technical repetitions. The data were analyzed using the OriginPro software (OriginPro 2021 (9.8), OriginLab Corporation, Northampton, MA, USA).

### 3.2. Electron–Ion Interaction Potential (EIIP)/Average Quasi-Valence Number (AQVN)

Specific recognition and targeting between interacting biological molecules at a distance of >5 Å were determined by the AQVN (average quasi-valence number) and the EIIP (electron–ion interaction potential) was derived from the general model pseudopotential [29], as follows:

EIIP = 0.25(Z*/(2π))Sin(1.04πZ*) where Z* is the AQVN determined by the following:

$$Z*=\frac{1}{N}\sum_{i=1}^{m}n_{i}Z_{i}$$

where *Z_i_* is the valence number of the *i*-th atomic component, *n_i_* is the number of atoms of the *i*-th component, *m* is the number of atomic components in the molecule, and *N* is the total number of atoms. The Z* and *EIIP* values are expressed in Rydberg units (Ry). AQVN and EIIP are unique physical properties that characterize, among the molecular descriptors, the long-range interactions between biological molecules [14]. The EIIP and AQVN of organic molecules have been shown to correlate strongly with their biological activity (mutagenicity, carcinogenicity, antibiotic activity, etc.) [30,31].

### 3.3. Unconstrained Conformational Search

**Figure 2:**
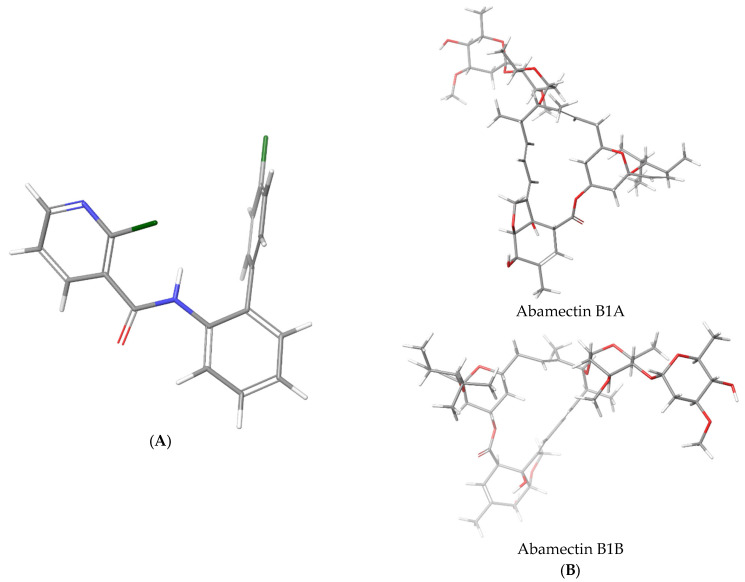
Structures of investigated pesticides: (**A**) boscalid and (**B**) abamectin (abamectins B1A and B1B).

Conformational analysis of the selected pesticides (boscalid and abamectins B1A and B1B) (Figure 2) was performed using MacroModel under Schrodinger Suite 2022-3 and Maestro v. 13.3 as an interface. Chloroform was used as the solvent. The conditions for the simulations were taken from previously published work [14]. Minimizations were initially carried out using charges derived from the MMFFs force field. The cut-off was extended, and the minimization method employed was Truncated Newton Conjugate Gradient (TNCG). A maximum of 10,000 iterations was allowed, with gradient convergence set to a threshold of 0.05. For the conformational search, torsion sampling was performed using the Monte Carlo Multiple Minimum (MCMM) method, with automatic settings applied during the calculation. The torsion sampling options were set to intermediate. The maximum number of steps was limited to 10,000, with 100 steps for each rotatable bond. For each search, 100 structures were stored, with an energy window of 21 kJ/mol for storing structures and a maximum atom deviation cut-off of 0.5 Å.

### 3.4. Molecular Docking Studies

Molecular docking studies with the selected pesticides (boscalid and abamectins B1A and B1B) were performed using human AChE as a target. In addition, the molecular docking of the selected pesticides to the binding site of donepezil in human AChE was performed [32]. In all cases, the crystal structure was obtained from the Protein Data Bank (entry 4EY7 [32]). AChE alone was prepared for docking using the Protein Preparation and Refinement Tool of Schrodinger Suite 2022-3. Previously optimized structures of the selected pesticides in MacroModel were ligands in the molecular docking studies. Molecular docking was performed with Glide under Schrodinger Suite 2022-3.

Human BChE (entry 7AWG) [33] as a target was also used in the molecular docking studies of boscalid and abamectins B1A and B1B considering the whole structure and a binding site of the compound (2-((1-(benzenesulfonyl)-1*H*-indol-4-yl)oxy)ethyl)(benzyl) amine with IC_50_ = 0.473 µM [33]. The enzyme alone was prepared for docking using the Protein Preparation and Refinement Tool of Schrodinger Suite 2022-3. Previously optimized structures of the selected pesticides in MacroModel were ligands in the molecular docking studies. Molecular docking was performed with Glide under Schrodinger Suite 2022-3.

### 3.5. ADMET In Silico Studies

The absorption, distribution, metabolism, elimination, and toxicity (ADMET) parameters of the selected pesticides (boscalid and abamectins B1A and B1B) were calculated using QikProp v7.0 software in normal mode (Schrödinger, Inc., New York, NY, USA).

ACD/Percepta (Advanced Chemistry Development, Inc., Toronto, ON, Canada) was also used to generate the ADMET profiles of the selected pesticides based on the SMILES strings of the compounds.

### 3.6. Metadynamics and Molecular Dynamics Simulations

To determine the most favorable docking conformation of boscalid within hBChE, we employed metadynamics simulations using Desmond molecular dynamics software (version 2018.4). This approach enhanced the exploration of ligand conformations by introducing biasing potentials, allowing the system to overcome energy barriers and sample alternative binding poses. The system was solvated with the simple point charge (SPC) water model, and chloride ions (Cl^−^) were added to maintain charge neutrality. Before the simulation, a six-step relaxation protocol was applied to equilibrate the system. The simulation was conducted under an NPT ensemble, with a Nosé–Hoover thermostat maintaining a stable temperature of 300 K and a Martyna–Tobias–Klein barostat regulating the pressure at 1.01325 bar. The metadynamics results revealed that boscalid’s binding pose within the active site was more thermodynamically stable than the whole-structure docking pose. This conclusion was based on the sampling frequency of ligand conformations along the collective variable trajectory, which showed a higher probability of boscalid remaining in the active site. This suggested that the active site conformation corresponded to a lower energy minimum compared to the alternative pose. To further investigate the stability and key interactions of boscalid, molecular dynamics simulations were performed under identical conditions. The system, containing approximately 52,000 atoms, underwent a 100 ns production run in the NPT ensemble. Atomic coordinate data and system energies were recorded at 1 ps intervals. To assess the stability and flexibility of the boscalid–hBChE complex, RMSD and RMSF analyses were conducted. These analyses provided insights into the structural fluctuations, conformational stability, and key ligand–protein interactions throughout the simulation.

## 4. Conclusions

Our combined ADMET and drug safety profiling showed that boscalid, despite having a better Glide score than donepezil, is a weak central nervous system penetrant. These features speak against its use as a candidate for the symptomatic treatment of Alzheimer’s disease. However, its non-mutagenicity and low toxicity (>5000 mg/kg), together with its ability to moderately inhibit human BChE, suggest that it can be redesigned in order to achieve better penetration properties to the central nervous system and better cholinesterase inhibitory properties. Docking and molecular dynamics studies revealed that boscalid predominantly stabilizes within the active site of hBChE through π-π stacking with Trp82 and interactions with His438, a key catalytic residue involved in ligand binding. This suggests that further modifications could enhance its affinity and inhibitory potency. Abamectins B1A and B1B were found to be non-penetrating to the CNS and non-mutagenic compounds.

## Figures and Tables

**Table 1:** Global minima and repeats of selected pesticides from the conformational search results and Glide scores obtained in molecular docking to human acetylcholinesterase (hAChE) and human recombinant butyrylcholinesterase (hBChE).

Compound	Global Minimum (kJ/mol)	Repeats	Glide Score (kJ/mol)			
			hAChE		hBChE	
			Whole Structure	Binding Site	Whole Structure	Binding Site
Boscalid	180.8	57	−21.3	−20.3	−27.2	−28.8
Abamectin B1A	179.0	61	−18.5	-	-	-
Abamectin B1B	172.8	58	−24.0	-	-	-

**Table 2:** In vitro inhibition of human acetylcholinesterase (hAChE), horse serum butyrylcholinesterase (hsBChE), and human recombinant butyrylcholinesterase (hBChE) by boscalid and abamectins B1A and B1B.

Compound	hAChE		hsBChE		hBChE	
	IC_50_ (µM)	*K_i_* (µM)	IC_50_ (µM)	*K_i_* (µM)	IC_50_ (µM)	*K_i_* (µM)
Abamectin (B1A + B1B)	/	*n.d.*	/	*n.d.*	/	*n.d.*
Boscalid	/	*n.d.*	/	*n.d.*	308.8 ± 12.6	*n.d.*
Neostigmine	4.8 ± 1.4	*n.d.*	62.8 ± 6.3	*n.d.*	173.5 ± 9.2	*n.d.*

IC_50_ was determined as the concentration of the compound inducing 50% inhibition of the enzyme activity. *K_i_*: inhibition constant. /: no activity for abamectin (IC_50_ > 72.2 µM) or boscalid (IC_50_ > 364.2 µM), *n.d.*: non-determined; data are the IC_50_ ± standard error (SE) of three independent measurements.

**Table 3:** Calculated absorption, distribution, metabolism, elimination, and toxicity (ADMET) parameters of the compounds.

Compound	MW	DM	MV	DHB	AHB	PSA	logP	logS	PCaco	PM	%HOA	VRF	VRT
Boscalid	343.2	5.7	993.1	1	3.5	42.2	4.6	−5.4	3029	2	100	0	0
Abamectin B1A	873.1	8.1	2515.3	2	18.5	142.4	6.3	−8.3	808	12	77	3	2
Abamectin B1B	859.1	8.0	2486.7	2	18.6	141.2	6.1	−8.0	724	12	75	3	2

**MW**: Molecular weight; **DM**: computed dipole moment; **MV**: total solvent-accessible volume; **DHB**: estimated number of hydrogen bond donors; **AHB**: estimated number of hydrogen bond acceptors; **PSA**: van der Waals surface area of polar nitrogen and oxygen atoms and carbonyl carbon atoms; **logP**: predicted octanol/water partition coefficient; **logS**: predicted aqueous solubility; **PCaco**: predicted apparent Caco-2 cell permeability; **PM**: number of likely metabolic reactions; **%HOA**: predicted human oral absorption percentage; **VRF**: number of violations of Lipinski rule of five (the rules are as follows: MW < 500, log P < 5, DHB ≤ 5, AHB ≤ 10, positive PSA value); **VRT**: number of violations of Jorgensen rule of three (the rules are as follows: log S > −5.7, PCaco > 22 nm/s, PM < 7).

**Table 4:** ADME and drug safety profiling of the selected pesticides using Percepta.

Pesticides	Caco-2 (cm/s)	PPB (%)	CNS	HIA (%)	Metabolic Stability	p-gp Substrate	CYP1A2 Inhibitor	CYP2C9 Inhibitor	CYP2C19 Inhibitor	CYP2D6 Inhibitor	CYP3A4 Inhibitor	Ames	hERG
Boscalid	86 × 10^−6^	99	−3.21	100	0.46	0.35	0.58	0.45	0.49	0.42	0.51	0.31	0.39
Abamectin B1A	4 × 10^−6^	97	−4.89	100	0.61	0.95	0.02	0.13	0.11	0.08	0.56	0.13	0.59
Abamectin B1B	6 × 10^−6^	97	−4.64	100	0.60	0.94	0.02	0.13	0.11	0.08	0.55	0.13	0.55

**Caco-2**: predicted apparent Caco-2 cell permeability (Pe > 7.0 × 10^−6^ cm/s); **PPB**: estimated plasma protein binding (%PPB > 90%); **CNS**: estimated central nervous system penetration (−3.5 ≤ CNS Score < −3.0, Log(PS x fu, brain) = −3.08, LogBB = −0.22); **HIA**: estimated human intestinal absorption (%HIA ≥ 70%); **Ames**: predicted ability of a chemical or drug to induce mutations in DNA (Score(positive Ames test) =Score(positive Ames test) < 0.33, Probability(positive Ames test) = 0.17 RI = 0.59); **hERG**: predicted cardiotoxic effects (Score(hERG IC_50_ < 10 μM) 0.33..0.67, Probability(hERG IC_50_ < 10 μM) = 0.16 RI = 0.31).

## Data Availability

The raw data supporting the conclusions of this article will be made available by the authors on request.

## Supplementary Materials

The following supporting information can be downloaded at: https://www.mdpi.com/article/10.3390/ijms26072865/s1.
