# Supplementary material for: Inhibitory Potential of Boscalid and Abamectin Towards Acetylcholinesterase and Butyrylcholinesterase: Computational and In Vitro Studies
Source: Int J Mol Sci. 2025 Mar 21;26(7):2865. doi: 10.3390/ijms26072865 (PMC11988634; doi:10.3390/ijms26072865)
Supplement: Supplementary file 1 [file ijms-26-02865-s001.zip › ijms-3477792-supplementary.pdf]

Supplementary Materials

Inhibitory Potential of Boscalid and Abamectin Towards Acetylcholinesterase and Butyrylcholinesterase: Computational and In Vitro Studies

Biljana Arsić [1,*], Stefan Petrović [1], Budimir S. Ilić [2], Milka Vrecl [3], Tomaž Trobec [3], Kristina Sepčić [4], Robert Frangež [3], Sanja M. Glišić [5] and Jelena S. Milićević [5,*]

1 Department of Chemistry, Faculty of Sciences and Mathematics, University of Niš, 18000 Niš, Serbia; stefan.petrovic@pmf.edu.rs
2 Department of Chemistry, Faculty of Medicine, University of Niš, 18000 Niš, Serbia; budimir.ilic@medfak.ni.ac.rs
3 Institute of Preclinical Sciences, Veterinary Faculty, University of Ljubljana, 1000 Ljubljana, Slovenia; milka.vreclfazarinc@vf.uni-lj.si (M.V.); tomaz.trobec@vf.uni-lj.si (T.T.); robert.frangez@vf.uni-lj.si (R.F.)
4 Department of Biology, Biotechnical Faculty, University of Ljubljana, 1000 Ljubljana, Slovenia; kristina.sepcic@bf.uni-lj.si
5 Department for Bioinformatics and Computational Chemistry, Institute of Nuclear Sciences, University of Belgrade, 11000 Belgrade, Serbia; sanja@vin.bg.ac.rs
* Correspondence: biljana.arsic@pmf.edu.rs or ba432@ymail.com (B.A.); jdjordjevic@vinca.rs (J.S.M.)

XYZ coordinates of a global minimum of boscalid:

Cl	2.54189	4.32663	-2.30984
Cl	-1.14513	1.38676	1.61217
O	-2.25683	-2.47186	0.44090
N	-0.51145	-1.06292	-0.21320
N	-3.42906	2.02282	0.64326
C	1.85470	-1.41174	-0.70444
C	0.60880	-1.93391	-0.27093
C	2.03355	-0.00535	-1.09877
C	2.97219	-2.26751	-0.76802
C	0.52785	-3.28645	0.08507
C	2.27515	0.98433	-0.13232
C	1.96121	0.37345	-2.44918
C	2.87123	-3.61041	-0.41090
C	1.65002	-4.11777	0.01539
C	-1.80867	-1.36909	0.14783
C	-2.76503	-0.24270	0.10086
C	2.43119	2.32220	-0.50542
C	2.11931	1.71073	-2.82406

C	2.35209	2.67927	-1.84981
C	-3.98610	-0.44170	-0.55417
C	-2.54765	0.99300	0.69443
C	-4.91230	0.59688	-0.61694
C	-4.58955	1.80090	-0.01267
H	3.93515	-1.88126	-1.09868
H	-0.40005	-3.73256	0.42764
H	-0.32037	-0.10210	-0.47716
H	2.34029	0.71516	0.92095
H	1.77900	-0.37631	-3.21753
H	3.74340	-4.25699	-0.46458
H	1.56370	-5.16459	0.29720
H	2.61364	3.07560	0.25646
H	2.05988	1.98663	-3.87363
H	-4.21363	-1.40115	-1.01445
H	-5.86360	0.46824	-1.12347
H	-5.27936	2.64100	-0.03666

XYZ coordinates of a global minimum of abamectin B1A:

C	14.72121	-21.24122	6.48837
C	14.83800	-21.31488	8.01463
C	14.84056	-19.92790	8.68780
C	13.57912	-19.13834	8.30810
C	14.92941	-20.01643	10.22726
C	16.23580	-20.63657	10.76898
C	16.09904	-20.92708	12.26452
C	14.90903	-20.92820	12.88264
C	13.59540	-20.68447	12.14140
O	13.81621	-20.76406	10.72879
C	12.56479	-21.73931	12.56689
C	11.20857	-21.42686	11.92687
C	10.81077	-20.00224	12.30930
C	11.90919	-19.01581	11.89842
O	13.13985	-19.38572	12.53279
C	11.53537	-17.59775	12.34921
C	10.26069	-17.12629	11.63840
C	9.13886	-16.67257	12.23772

C	7.83836	-16.31875	11.49763
C	6.75376	-17.37353	11.79388
C	7.14714	-18.76118	11.27848
C	6.94309	-19.88527	11.98173
C	7.34953	-21.21570	11.47536
C	7.08384	-22.36715	12.11312
C	6.26471	-22.51989	13.38876
O	6.12805	-23.93029	13.51532
C	6.29611	-24.48978	12.21458
C	7.50055	-23.74499	11.62764
C	8.80498	-24.19280	12.31798
C	8.95148	-25.70853	12.20483
C	7.87897	-26.51353	12.19110
C	6.43348	-26.02247	12.24985
O	5.84670	-26.49116	13.44222
C	8.09511	-28.02564	12.11233
C	10.03943	-23.46365	11.77680
O	10.77054	-23.87983	10.91659
O	10.18739	-22.30395	12.42465
O	7.54474	-23.84175	10.21454
C	5.42381	-16.98058	11.14196
O	8.04168	-16.22064	10.07890
C	8.65164	-14.98563	9.68119
C	9.17202	-15.08149	8.24470
C	8.00630	-15.18942	7.25871
C	7.10413	-13.95993	7.45981
C	6.61691	-13.96010	8.91969
O	7.73149	-13.89654	9.81926
C	5.70614	-12.76721	9.21601
O	5.98613	-14.02859	6.56202
C	6.13436	-13.35187	5.30813
C	5.22092	-13.95524	4.23335
C	3.75661	-13.64240	4.54629
C	3.63913	-12.12211	4.62880
C	4.55207	-11.60141	5.74417
O	5.91884	-11.94425	5.47481

C	4.44932	-10.07816	5.85611
O	2.26800	-11.82340	4.87150
O	2.85241	-14.08077	3.51756
C	2.59672	-15.47541	3.55584
O	8.49855	-15.23089	5.90992
C	8.81160	-16.55079	5.45046
C	9.11807	-16.46535	13.75520
C	17.43320	-19.70276	10.55473
H	14.86820	-19.00158	10.62345
H	16.44160	-21.58920	10.28401
H	11.28253	-21.46976	10.83822
H	12.01999	-19.01630	10.81426
H	7.47604	-15.36135	11.87391
H	6.59285	-17.41369	12.87221
H	5.40345	-24.24584	11.63323
H	8.74158	-23.97753	13.38610
H	5.90173	-26.44059	11.39397
H	9.50013	-14.76668	10.33044
H	7.42394	-16.08818	7.46926
H	7.67980	-13.05076	7.27651
H	6.04502	-14.86792	9.11026
H	7.15677	-13.45458	4.94802
H	3.47241	-14.07559	5.50698
H	3.92894	-11.68165	3.67340
H	4.23609	-12.03490	6.69096
H	14.87317	-22.23691	6.07048
H	13.73121	-20.89324	6.19457
H	15.48052	-20.56922	6.08734
H	14.01385	-21.91654	8.40022
H	15.77372	-21.82646	8.23357
H	15.69607	-19.36228	8.31961
H	12.68731	-19.72746	8.52335
H	13.53866	-18.20809	8.87515
H	13.59477	-18.88474	7.24872
H	16.98888	-21.12387	12.84469
H	14.87078	-21.11587	13.94613

H	12.90343	-22.72865	12.25642
H	12.45611	-21.72826	13.65269
H	9.87933	-19.75650	11.80179
H	10.65335	-19.94570	13.38756
H	11.43445	-17.61173	13.43228
H	12.34801	-16.92037	12.08432
H	10.24443	-17.25570	10.56590
H	7.62738	-18.81855	10.31181
H	6.46310	-19.80692	12.94693
H	7.89477	-21.27245	10.54266
H	6.78606	-22.10765	14.25336
H	5.28683	-22.05182	13.26778
H	9.94886	-26.12306	12.15010
H	5.85320	-25.74182	14.05003
H	7.60212	-28.41436	11.22106
H	9.15449	-28.27992	12.06824
H	7.65735	-28.49753	12.99253
H	7.94503	-24.68706	9.98876
H	4.64764	-17.68425	11.44414
H	5.13623	-15.97854	11.46114
H	5.51353	-17.00148	10.05584
H	9.75497	-14.18803	8.01631
H	9.82154	-15.95302	8.16022
H	4.76275	-12.88644	8.68666
H	5.49904	-12.72484	10.28556
H	6.18826	-11.83979	8.90588
H	5.48161	-13.53186	3.26225
H	5.38342	-15.03258	4.20131
H	3.42567	-9.79021	6.09670
H	5.11156	-9.72653	6.64749
H	4.74086	-9.61607	4.91271
H	1.77179	-12.38282	4.26428
H	1.87776	-15.71932	2.77347
H	3.50809	-16.04509	3.37645
H	2.17222	-15.76020	4.51939
H	9.20825	-16.48516	4.43718

H	9.56096	-17.01683	6.09021
H	7.91237	-17.16787	5.43453
H	8.38334	-15.70061	14.00750
H	8.83695	-17.39699	14.24702
H	10.08009	-16.12151	14.13204
H	18.32958	-20.14785	10.98771
H	17.60953	-19.54806	9.49118
H	17.24815	-18.74020	11.03284

XYZ coordinates of a global minimum of abamectin B1B:

C	-15.21078	-12.23764	-15.42864
C	-14.71596	-11.75236	-14.06195
C	-14.00023	-12.91726	-13.37157
C	-14.58239	-13.67650	-12.43113
C	-13.86705	-14.78542	-11.76014
C	-14.44489	-15.61777	-10.87898
C	-15.91708	-15.63032	-10.48644
O	-16.02820	-16.80764	-9.69559
C	-14.95866	-17.67227	-10.07195
C	-13.73470	-16.75383	-10.16287
C	-13.28229	-16.31097	-8.75650
C	-13.01939	-17.54099	-7.89063
C	-13.72167	-18.67174	-8.05399
C	-14.80587	-18.86621	-9.11261
O	-16.03905	-19.07065	-8.46208
C	-13.43424	-19.85619	-7.12994
C	-12.06837	-15.37640	-8.79411
O	-10.92262	-15.72035	-8.66513
O	-12.48397	-14.11852	-8.97161
C	-11.45861	-13.13745	-9.18623
C	-12.08368	-11.99320	-9.98260
C	-11.07321	-10.85494	-10.16120
C	-11.73194	-9.68022	-10.89609
C	-12.15057	-10.09777	-12.31128
C	-13.38889	-9.98641	-12.83824
C	-13.79903	-10.52309	-14.21952
O	-12.65618	-10.87208	-15.01692

C	-12.01188	-9.73476	-15.60588
C	-10.62251	-10.11329	-16.12535
C	-10.73774	-11.06395	-17.31935
C	-11.58143	-10.36445	-18.39860
C	-12.95881	-10.04213	-17.79237
O	-12.81581	-9.18164	-16.65453
C	-13.87364	-9.34134	-18.79851
O	-11.72758	-11.23034	-19.53415
C	-10.77141	-11.06452	-20.58789
C	-10.61647	-12.34888	-21.41250
C	-11.88450	-12.61162	-22.22681
C	-12.11068	-11.37894	-23.09931
C	-12.29021	-10.14825	-22.20385
O	-11.11988	-9.93660	-21.40152
C	-12.52726	-8.89605	-23.05190
O	-13.26259	-11.63851	-23.89549
O	-11.76498	-13.73919	-23.11132
C	-11.89376	-14.98598	-22.44736
O	-9.43333	-11.37766	-17.83255
C	-8.81588	-12.49982	-17.19167
C	-14.48936	-9.27040	-12.04928
O	-10.63989	-10.40022	-8.87316
C	-9.97246	-11.41912	-8.12227
C	-10.94301	-12.57810	-7.85656
C	-9.56019	-10.78431	-6.79528
C	-8.28605	-10.48677	-6.50190
C	-7.14852	-10.72067	-7.49747
C	-7.71783	-10.98676	-8.90759
O	-8.81102	-11.90526	-8.80429
C	-6.68524	-11.52509	-9.91966
C	-7.32529	-11.63283	-11.30963
C	-6.11585	-12.89814	-9.53312
C	-6.22448	-9.49690	-7.48660
O	-12.69716	-17.33057	-10.93684
H	-15.59109	-11.46112	-13.47918
H	-15.18778	-18.06658	-11.06510

H	-14.09496	-15.77004	-8.26845
H	-14.55090	-19.75233	-9.69561
H	-10.63207	-13.53918	-9.77606
H	-10.22535	-11.20109	-10.75216
H	-14.37177	-9.75081	-14.73430
H	-11.88710	-8.95188	-14.85692
H	-11.24786	-11.98012	-17.01656
H	-11.09581	-9.43274	-18.69470
H	-13.44723	-10.96841	-17.49080
H	-9.79204	-10.83720	-20.16984
H	-12.74160	-12.74245	-21.56376
H	-11.25379	-11.23772	-23.76004
H	-13.15971	-10.29828	-21.56696
H	-6.59119	-11.58599	-7.14185
H	-8.09871	-10.03943	-9.29244
H	-15.93374	-13.04203	-15.29010
H	-15.69245	-11.41729	-15.96125
H	-14.37700	-12.61292	-16.02203
H	-12.97436	-13.11109	-13.65157
H	-15.60816	-13.46573	-12.16369
H	-12.81952	-14.93842	-11.98304
H	-16.18476	-14.75020	-9.90076
H	-16.54421	-15.70822	-11.37562
H	-12.25730	-17.47457	-7.12618
H	-16.50490	-18.22716	-8.51179
H	-13.12895	-20.71467	-7.72853
H	-12.64433	-19.62695	-6.41413
H	-14.34120	-20.11242	-6.58147
H	-12.38956	-12.37672	-10.95466
H	-12.96331	-11.62132	-9.45478
H	-12.56973	-9.34067	-10.29090
H	-11.00901	-8.86763	-10.97635
H	-11.39538	-10.60288	-12.89593
H	-10.09895	-9.20780	-16.43555
H	-10.05867	-10.58378	-15.31957
H	-14.15523	-10.03881	-19.58505

H	-14.77984	-9.00592	-18.29362
H	-13.36476	-8.48088	-19.23365
H	-9.76794	-12.24014	-22.08957
H	-10.41929	-13.17842	-20.73346
H	-13.43312	-9.01470	-23.64691
H	-12.64344	-8.03127	-22.39834
H	-11.67896	-8.73183	-23.71670
H	-13.14880	-12.53566	-24.22723
H	-11.82973	-15.78332	-23.18808
H	-11.09461	-15.12602	-21.71999
H	-12.85981	-15.05417	-21.94554
H	-8.71691	-12.32846	-16.11982
H	-9.40361	-13.40253	-17.36295
H	-7.82229	-12.64402	-17.61638
H	-15.22617	-8.86266	-12.74150
H	-14.98573	-9.98113	-11.38797
H	-14.09738	-8.43535	-11.47070
H	-11.79024	-12.21925	-7.26976
H	-10.43267	-13.36244	-7.29600
H	-10.32891	-10.57654	-6.06483
H	-8.05924	-10.05552	-5.53770
H	-5.85954	-10.81834	-9.99950
H	-8.11606	-12.38336	-11.30496
H	-7.74531	-10.66960	-11.60048
H	-6.57027	-11.92047	-12.04173
H	-6.92285	-13.61628	-9.38480
H	-5.46533	-13.26014	-10.32961
H	-5.52472	-12.82775	-8.62143
H	-5.86958	-9.31227	-6.47220
H	-5.35875	-9.66802	-8.12486
H	-6.76375	-8.61727	-7.83985
H	-12.21139	-17.93980	-10.37239
